# PD-L1 expression heterogeneity in non-small cell lung cancer: evaluation of small biopsies reliability

**DOI:** 10.18632/oncotarget.21485

**Published:** 2017-10-04

**Authors:** Enrico Munari, Giuseppe Zamboni, Marcella Marconi, Marco Sommaggio, Matteo Brunelli, Guido Martignoni, George J. Netto, Francesca Moretta, Maria Cristina Mingari, Matteo Salgarello, Alberto Terzi, Vincenzo Picece, Carlo Pomari, Gianluigi Lunardi, Alberto Cavazza, Giulio Rossi, Lorenzo Moretta, Giuseppe Bogina

**Affiliations:** ^1^ Department of Pathology, Sacro Cuore Don Calabria Hospital, Negrar, Italy; ^2^ Department of Pathology AOUI, University of Verona, Verona, Italy; ^3^ Department of Pathology, Pederzoli Hospital, Peschiera del Garda, Italy; ^4^ Department of Pathology, The University of Alabama at Birmingham, Birmingham, AL, USA; ^5^ Department of Laboratory Medicine, Sacro Cuore Don Calabria Hospital, Negrar, Italy; ^6^ Department of Experimental Medicine (DIMES), University of Genoa, Genoa, Italy; ^7^ Department of Nuclear Medicine, Sacro Cuore Don Calabria Hospital, Negrar, Italy; ^8^ Department of Thoracic Surgery, Sacro Cuore Don Calabria Hospital, Negrar, Italy; ^9^ Department of Oncology, Sacro Cuore Don Calabria Hospital, Negrar, Italy; ^10^ Department of Pulmonology, Sacro Cuore Don Calabria Hospital, Negrar, Italy; ^11^ Department of Pathology, Arcispedale S. Maria Nuova/IRCCS, Reggio Emilia, Italy; ^12^ Department of Pathology, Azienda USL Valle d'Aosta, Aosta, Italy; ^13^ Immunology Research Area, IRCCS Bambino Gesu Pediatric Hospital, Rome, Italy

**Keywords:** PD-L1, lung cancer, immunotherapy, SP263, pembrolizumab

## Abstract

Immunotherapy with checkpoint inhibitors, allowing recovery of effector cells function, has demonstrated to be highly effective in many tumor types and represents a true revolution in oncology. Recently, the anti-PD1 agent pembrolizumab was granted FDA approval for the first line treatment of patients with advanced non–small cell lung cancer (NSCLC) whose tumors show PD-L1 expression in ≥ 50% of neoplastic cells and as a second line treatment for patients with NSCLC expressing PD-L1 in ≥1% of neoplastic cells, evaluated with a validated assay. For the large majority of patients such evaluation is made on small biopsies. However, small tissue samples such as core biopsies might not be representative of tumors and may show divergent results given the possible heterogeneous immunoexpression of the biomarker. We therefore sought to evaluate PD-L1 expression concordance in a cohort of 239 patients using tissue microarrays (TMA) as surrogates of biopsies stained with a validated PD-L1 immunohistochemical assay (SP263) and report the degree of discordance among tissue cores in order to understand how such heterogeneity could affect decisions regarding therapy.

We observed a discordance rate of 20% and 7.9% and a Cohen's κ value of 0.53 (moderate) and 0,48 (moderate) for ≥ 1% and ≥ 50% cutoffs, respectively.

Our results suggest that caution must be taken when evaluating single biopsies from patients with advanced NSCLC eligible for immunotherapy; moreover, at least 4 biopsies are necessary in order to minimize the risk of tumor misclassification.

## INTRODUCTION

Programmed cell death 1 (PD1) is an inhibitory receptor originally identified in T lymphocytes [1, 2] that, upon interaction with its ligand(s) PD-L1, delivers inhibitory signals that downregulate T cell function. While, under physiological conditions, this interaction leads to peripheral T-cell tolerance, in cancer patients it may impair T cell responses against tumor cells. Very recently, PD1 expression has been documented also in NK cells, thus suggesting a further impairment of immune response, particularly against tumors resistant to T-cell activity, because of loss of HLA-I molecules [3].

Immunotherapy with checkpoint inhibitors has been shown to be highly effective in many tumor types and represents a revolution in oncology [4]. Currently, there are four drugs targeting the PD1/PD-L1 axis which have been approved by the Food and Drug Administration (FDA): two against PD-L1 (atezolizumab and durvalumab) and two against PD1 (nivolumab and pembrolizumab).

Predicting which patients will respond to checkpoint inhibitors therapy is a major issue and so far has been mainly based on the immunohistochemical evaluation of PD-L1 expression on tumor cells [5, 6]. Although some studies found a significant correlation between expression of PD-L1 and response to therapy [7–11], others have not; specifically, responses have been observed in patients whose tumors lacked PD-L1 expression [12, 13].

Recently, anti PD1 agent pembrolizumab was granted FDA approval after clinical trials that were conducted in patients with advanced lung adenocarcinoma or squamous carcinoma on the basis of PD-L1 immunoexpression on viable tumor cells [10]. Specifically, pembrolizumab is indicated for the first line treatment of patients with advanced non–small cell lung cancer (NSCLC) whose tumors show PD-L1 expression in ≥ 50% of neoplastic cells and as a second line treatment for patients with NSCLC expressing PD-L1 in ≥1% of neoplastic cells, evaluated with a validated assay. In this setting, the immunohistochemical evaluation of PD-L1 expression on tumor specimens has become critical; moreover, it is necessary to keep in mind that for the large majority of patients, such evaluation is made on small biopsies. However, small tissue samples like core biopsies might not be representative of tumor specimen and display divergent results because of the possible heterogeneous immunoexpression of the biomarker. Specifically, if only one random biopsy would be available, it could be possible that a proportion of cases might be misclassified. Therefore, it is of major importance to understand the magnitude of this problem as it can profoundly impact on the patient's management; in this regard, only a few studies have been conducted, reporting conflicting results. For these reasons, in this study we assessed the heterogeneity of PD-L1 expression in NSCLC using tissue microarrays as surrogate of small biopsies with a validated immunohistochemical assay (Ventana's SP263) in order to understand its impact in patient selection for therapy in first and second line setting.

## RESULTS

### Patients characteristics

From an initial 241 patients, two were discarded from the analysis because of failure in TMA construction. Overall 239, patients were included in this study; of these, 172 were males and 67 were females; median age was 71 years (range 41-87 years), for whom surgically resected specimen was available. Of the 239 patients, 159 were diagnosed with adenocarcinoma, 65 with squamous cell carcinoma, 9 with large cell carcinoma, 3 with adeno-squamous carcinoma, 2 with large cell neuroendocrine carcinoma and 1 with sarcomatoid carcinoma. The median size of the tumors was 3 cm (range 0.8-21 cm).

Lymph node status was available for 220 cases.

### PD-L1 expression and clinical-pathological features

Associations between PD-L1 expression and clinicopathological features are summarized in Table 1. We found that PD-L1 positive tumors tend to show a higher stage; moreover, when considering 1% cutoff, squamous cell carcinomas tend to be more often positive than adenocarcinomas.

**Table 1:** Associations between PD-L1 expression status and clinical-pathological parameters

Variable	PD-L1 ≥ 1%			PD-L1 ≥ 50%		
	Positive	Negative	P value	Positive	Negative	P value
**Patients**	93	146		29	210	
**Age**			0.54			0.82
< 71 y	44	75		14	106	
≥ 71 y	49	71		14	106	
**Sex**			0.13			0.34
Male	72	100		23	149	
Female	21	46		6	61	
**Histology**			0.06			0.59
ADC (159)	57	102		18	141	
SCC (65)	32	33		9	56	
**Diameter**			0.006			0.06
< 30 mm	34	80		9	105	
≥ 30 mm	59	66		20	105	
**N Stage (220)**			0.27			0.02
N0 (155)	57	98		14	141	
N1-N3 (65)	29	36		13	52	

ADC: adenocarcinoma; SCC: squamous cell carcinoma.

### PD-L1 expression within tissue cores

Overall, when considering a cutoff of ≥ 1% of cells stained by PD-L1, 93/239 (40%) of cases resulted positive and 146/239 (60%) were negative. Among positive cases, 45/93 (48%) showed full concordance between evaluable cores: specifically, 32 cases showed positivity in 5/5 cores, 7 cases in 4/4 cores and 6 in 3/3 cores.

Importantly, 48/93 cases (52%) showed discordant results in at least 1 core: of note, within the positive cases with all 5 cores available for evaluation, in 15 cases 1 core out of 5 resulted positive, in 9 cases 2 out of 5 cores were positive, in 13 cases 3 out of 5 cores were positive and in 4 cases positivity was seen in 4 out of 5 cores. Among cases with 4 cores evaluable, 2 cases were positive in 1 core and 2 cases showed positivity in 3 cores. Among cases with 3 cores available, 1 case was positive in 1 core and 2 showed positivity in 2 cores. Only 4 cases had less than 3 cores available for evaluation and none of these stained positive for PD-L1 (Table 2A).

**Table 2:** PD-L1 expression within tissue cores

A: PD-L1 Cutoff ≥ 1%								
		Tot	Neg			Positive		
**Number of evaluable cores**	**5**	191	118	15	9	13	4	32
	**4**	29	18	2	0	2	7	
	**3**	15	6	1	2	6		
	**2**	3	3	0	0			
	**1**	1	1	0				
		**239**	**0**	**1**	**2**	**3**	**4**	**5**
**Number of positive cores**								
**B: PD-L1 Cutoff ≥ 50%**								
		**Tot**	**Neg**			**Positive**		
**Number of evaluable cores**	**5**	191	169	6	4	3	4	5
	**4**	29	26	1	0	1	1	
	**3**	15	11	0	0	4		
	**2**	3	3	0	0			
	**1**	1	1	0				
		**239**	**0**	**1**	**2**	**3**	**4**	**5**
**Number of positive cores**								

Each cell shows number of total cases (239) relative to the number of positive cores out of the number of available cores using ≥ 1% (A) and ≥ 50% (B) cutoffs. If a single random biopsy was available, incorrect categorization might occur in up to 7.9% and 20% of patients with advanced NSCLC eligible for first (50% cutoff) and second (1% cutoff) line therapy with pembrolizumab, respectively.

When considering a cutoff of ≥ 50% of neoplastic cells expressing PD-L1, 29/239 cases (12%) were positive while 210/239 (88%) resulted negative. Of the positive cases, 10 (34%) showed 100% concordance between available cores: among these, 5 had all 5 cores available, 1 case had 4 cores available and 4 cases had 3 cores available. Importantly, 19 (66%) of the positive cases showed discordant results in the available cores: among cases with 5 cores available, 6 were positive in just 1 core, 4 were positive in 2 cores, 3 showed positivity in 3 cores and 4 had 4 cores positive. One case showed PD-L1 positivity in 1 out of 4 cores and 1 case stained positive in 3 out of 4 cores (Table 2B).

When considering the two main histotypes, namely adenocarcinoma and squamous cell carcinoma, positivity was seen in 57/159 cases (36%) and in 32/65 cases (49%) using a cutoff of ≥ 1% of cells, respectively. In positive adenocarcinomas, full concordance was seen in 26 cases (46%) while 31 cases (54%) showed discordant results within the cores.

At the same cutoff (≥ 1%) in squamous cell carcinomas, 100% concordance was seen in 16 of the 32 positive cases (50%) while the other half showed discordant results within the cores.

**Figure 1:**
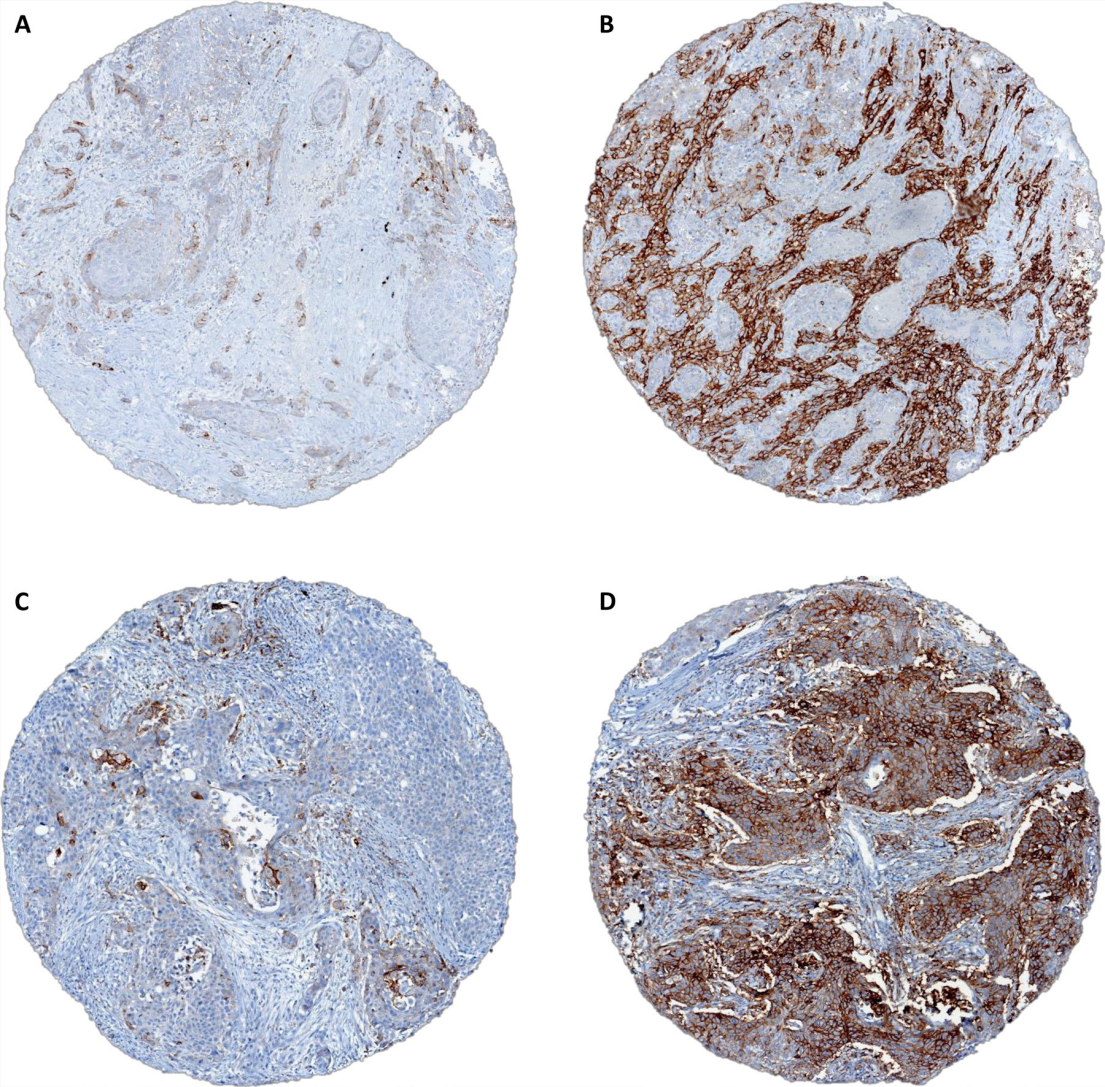
**(A, B)** discordant PD-L1 expression within tissue cores from a single adenocarcinoma case (2% vs 80%); **(C, D)** discordant PD-L1 expression within tissue cores from a single squamous cell carcinoma case (5% vs 70%).

When a cutoff of ≥ 50% of cells expressing PD-L1 was applied, 18/159 (11%) adenocarcinomas and 9/65 (14%) squamous cell carcinomas were positive. Among positive adenocarcinomas, concordance within all available cores was reached in 7 (39%) cases while 11 cases (61%) showed discordance within cores. Among positive squamous cell carcinomas, 3/9 cases (33%) showed full concordance, all with 5 cores available, while 6/9 cases (67%) showed discordant results (Supplementary Tables 1 and 2). Figure 1 shows representative discordant PD-L1 expression in a single case of adenocarcinoma (A and B) and squamous cell carcinoma (C and D).

Overall, considering all cases, we observed a discordance rate of 20% and 7.9% and a Cohen's κ value of 0,53 (moderate) and 0,48 (moderate) for ≥ 1% and ≥ 50% cutoffs, respectively.

### Whole sections evaluation and assessment of optimal number of cores

**Figure 2:**
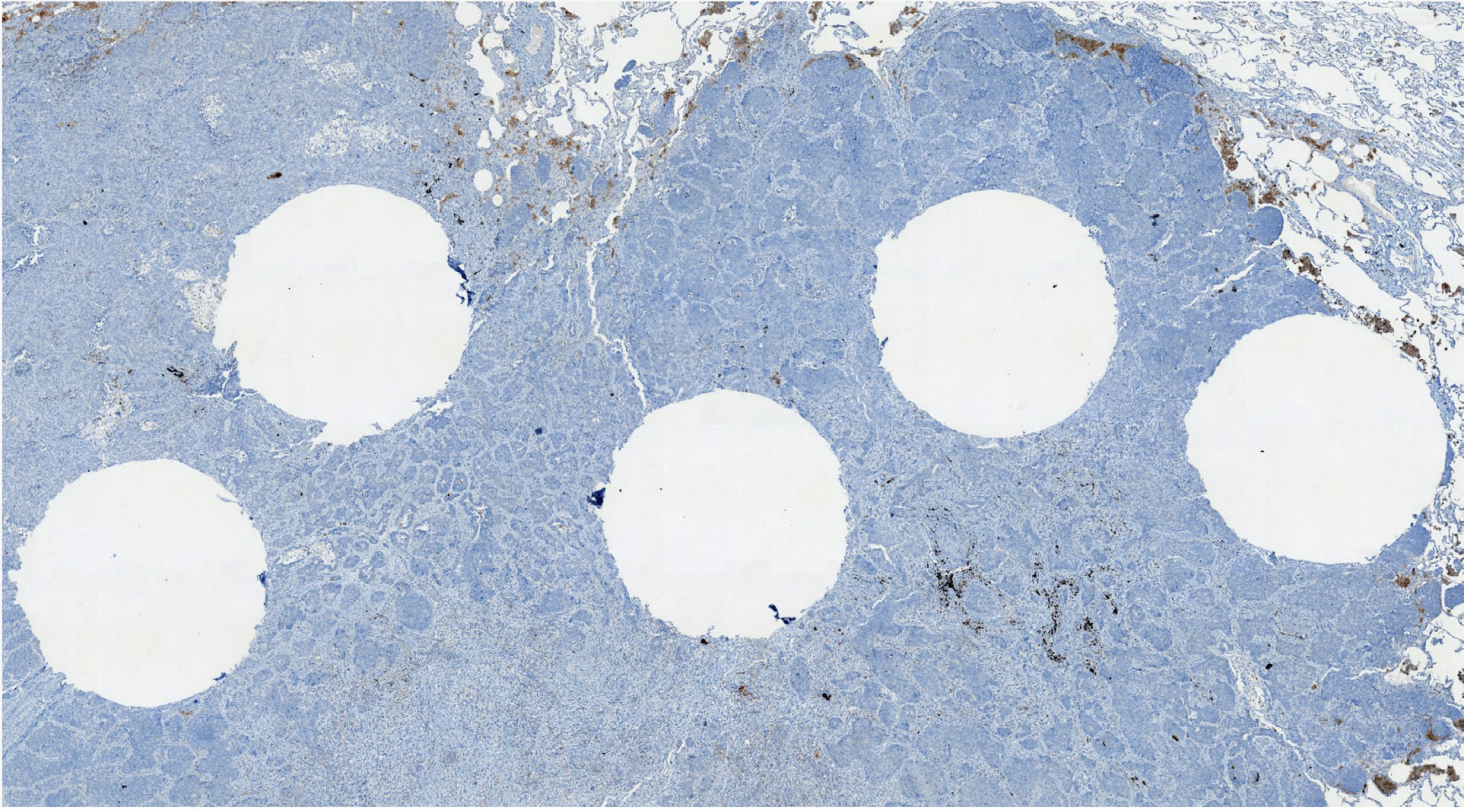
Representative whole section of a case negative for PD-L1 expression; white holes correspond to TMA cores

In order to account for possible false negative results among tissue cores, we randomly selected 30 cases among those with all 5 cores negative and stained corresponding whole section with PD-L1 (Figure 2). We found perfect correlation for all cases.

We then evaluated the optimal number of cores to reach the highest correlation, considering as gold standard the results obtained by analyzing cases with the maximum number of cores available (n=5). Overall, 191 cases had all 5 cores available and were used for the analysis.

We compared ROC curves obtained in this group of patients by one to four cores (cores were randomly ordered on the basis of their increasing number). Such analysis indicated statistically significant differences between gold standard and number of cores < 4 for both 1% and 50% cutoff; therefore, at least 4 biopsies are necessary to reach optimal correlation (Supplementary Table 3).

## DISCUSSION

Several studies indicated that PD-L1 expression on different tumor types correlates with better response to treatment with anti-PD1 or anti PD-L1 antibody [7–10]. On the other hand, other works reported that a significant percentage of patients whose neoplasms did not express PD-L1 benefited from the therapy [12, 13]. However, FDA recently granted approval for anti-PD1 pembrolizumab as a single agent for patients with tumors expressing PD-L1 in ≥ 50% and ≥ 1% of neoplastic cells for first and second line therapy, respectively. This was defined after the results of clinical trials, where, among patients with PD-L1 expressed in at least 50% of neoplastic cells the response rate was up to 44.8% [10, 11].

The vast majority of patients in these studies presented with advanced diseases and often only small biopsies were available. Therefore, it is crucial to understand if biopsies can indeed be reliable to accurately classify tumors according to PD-L1 expression, specifically at cutoffs of ≥ 1% and ≥ 50% of positivity in neoplastic cells. It is evident that this information is relevant for the therapeutic choice.

Karlsson and colleagues addressed the methodologic aspects of TMA construction relative to sample size (diameter and number of tissue cylinders) in order to account for tumor heterogeneity in lung cancer and other tumors and found that 0.6 mm cylinders are as informative as 1 mm cylinders and that 3 tissue cylinders (cores) for each case can fulfill a precision criterion of practical value [14].

Therefore we built our TMAs by randomly sampling 5 cores for each case included in the study.

Using 1% cutoff for positivity in neoplastic cells, of the 93/239 (39%) cases that were positive in at least 1 core, 45 (48%) showed full concordance between all available cores while 48 cases (52%) showed discordant results, with the majority of cases (15) showing 1 positive core out of 5, and 13 cases showing 3 cores positive out of 5. Using 50% cutoff for positivity in neoplastic cells, we found 29/239 (12%) cases to be positive in at least 1 core: among these, 10 (34%) cases showed full concordance between all available cores while the other 19 (66%) showed discordant results.

Cohen's κ value was 0,53 (moderate) and 0,48 (moderate) for ≥ 1% and ≥ 50% cutoffs, respectively.

These results mean that, in practice, if a single random biopsy was available, incorrect categorization might occur in up to 7.9% and 20% of patients with advanced NSCLC eligible for first and second line therapy with pembrolizumab, respectively. According to our analysis, at least 4 biopsies are necessary to reach optimal correlation.

Only a few studies have addressed the issue of the impact of small biopsies in determining eligibility for pembrolizumab treatment in NSCLC patients and reported conflicting results.

Kitazono et al. studied PD-L1 IHC expression in 79 paired biopsy and resected specimen of NSCLC cases (45 adenocarcinomas, 23 squamous cell carcinomas and 11 other types) using a polyclonal clone (number 4059; ProSci, Poway, CA); using ≥ 1% cutoff they found positivity in 38% of cases with 92.4% concordance (κ value 0,8366, almost perfect agreement) while using ≥ 50% cutoff 21.8% of cases resulted positive with concordance in 83.5% (κ value 0,3969, fair agreement) [15].

In another study, Ilie et al. evaluated PD-L1 IHC expression in 160 paired biopsy and resected specimen of NSCLC (33 squamous cell carcinomas and 127 adenocarcinomas) using SP142 clone (Ventana, Roche, Tucson, AZ); at ≥ 1% cutoff (considering PD-L1 expression on tumor cell only), the authors found positivity in 23% of resection specimen versus 7% positive biopsies with an overall discordance rate of 19% and a κ value of 0.396 (poor agreement) between resection specimen and biopsies [16].

Gniadek and colleagues evaluated PD-L1 expression on tissue microarrays from 150 NSCLC cases (71 adenocarcinomas and 79 squamous cell carcinomas) using SP142 antibody (Spring Bioscience, Pleasanton, CA) and Abcam detection kit: at ≥ 1% cutoff, 47% of cases were positive while at ≥ 50% cutoff they found positivity in 24% of cases. Of note, they found discrepancies among cores in 28 out of 71 (40%) positive cases in total [17].

Overall, positivity rate at ≥ 1% cutoff is comparable among our and previous works; however we register a somehow lower rate of positivity at ≥ 50% cutoff: this difference might be explained by our lower number of squamous cell carcinomas (which tend to present higher positivity rates) and the different clones and detection systems used in the different studies [18].

In fact, one very important issue regarding PD-L1 IHC testing is related to the type of anti PD-L1 antibody is used.

Currently, there are 4 validated assays for PD-L1: 2 are manufactured by Dako (Carpenteria, CA) and are optimized for use with the detection systems developed for the Dako Link 48 staining platform while the other 2 assays have been developed on the Ventana BenchMark platform. Each assay was developed with a unique primary antibody (clone) against PD-L1, namely, 28-8 (Dako) with nivolumab (Bristol-Myers Squibb), 22C3 (Dako) with pembrolizumab (Merck & Co., Inc.), SP263 (Ventana) with durvalumab (AstraZeneca), and SP142 (Ventana) with atezolizumab (Genentech). The availability of multiple approved PD-L1 IHC assays poses serious difficulties regarding the application of PD-L1 testing in terms of which clone and platform to be used. For these reasons, harmonization studies have been conducted and results showed 3 clones, specifically 22C3 (Dako), 28-8 (Dako) and SP263 (Ventana) to be comparable while SP142 stained fewer cells overall and resulted to be an outlier [19]. For this reason we chose SP263 (Ventana) for the evaluation and scoring of our specimens. Moreover, Ventana's SP263 application was recently expanded to include patients being considered for pembrolizumab immunotherapy.

Although PD-L1 expression on tumor cells has been shown to be correlated with response to anti-PD-1 axis targeted therapies, not all PD-L1-positive patients benefit from such therapies; on the other hand, some PD-L1-negative patients do respond. Therefore, precise PD-L1 quantification on tumor cells might not be the only variable to rely on in order to select patients for such therapies. It is reasonable to think that other molecules, like PD-L2, may also play an important role in regulating the immune response. In this context, Yearley et al. detected PD-L2 expression in different tumor types in the absence of PD-L1 and found PD-L2 expression to be predictive of progression free survival with pembrolizumab independent of PD-L1 status [20]. These results suggest that evaluation of both PD-L1 and PD-L2 will be necessary in the future.

Our work suffers from a number of limitations, including the fact that we did not use whole sections for all cases for the analysis of PD-L1 expression; however, we built our TMAs punching 5 cores for every case and this has been demonstrated to yield good precision compared with whole sections.

Moreover, we used only one PD-L1 clone (SP263) which, even though it was demonstrated to be comparable with Dako's 22C3 and 28-8 clones, might not perfectly match all possible cases.

Lastly, we did not evaluate the correspondence between primary tumors and metastasis.

In conclusion, we assessed the expression heterogeneity of PD-L1 in a large cohort of patients with NSCLC using tissue cores as surrogates of biopsies using two cutoffs (≥ 1% and ≥ 50% of cells) and found discordant results in a significant number of cases.

To our knowledge, this is the largest study so far addressing this issue with a validated assay.

Our results suggest that caution must be taken when evaluating single biopsies from patients with advanced NSCLC eligible for immunotherapy; according to the current analysis, at least 4 biopsies are necessary to reach optimal correlation. Further studies aiming at establishing the minimum number of biopsies to be taken in order to minimize the risk of tumor misclassification are needed.

## MATERIALS AND METHODS

### Study cohort

The study cohort consisted of consecutive patients with primary NSCLC who had undergone surgical resection at the Sacro Cuore Don Calabria Hospital of Negrar, Verona (Italy) between 2003 and 2017 with available slides and paraffin embedded tissue blocks.

Tumors were classified according to the 2012 WHO classification and staging was done using the TNM staging manual (7^th^ edition). Patients demographics and clinical data were retrieved from the digital archives.

Investigations have been conducted according to principles expressed in the Declaration of Helsinki.

### Tissue microarray construction

For every case, all H&E stained slides were reviewed for diagnosis confirmation; one block was then selected for tissue microarray (TMA) construction. It has been demonstrated that TMAs containing at least 3 cores per case yield satisfactory agreement compared with whole section in lung cancer [14]. Therefore, for each block, 5 cores with a diameter of 0,6 mm were obtained randomly from the diverse areas of the tumor. Overall, 11 TMAs were built.

### Whole sections analysis

In order to account for possible false negative results among tissue cores, 30 cases among those with all 5 cores negative have been randomly selected and corresponding whole section have been stained with PD-L1.

### Immunohistochemistry and scoring

From each block, 5μm sections were cut and stained with PD-L1 (clone SP263, Ventana) on an automated staining platform (Benchmark ULTRA; Ventana). An OptiView DAB IHC Detection Kit (Ventana) and an OptiView Amplification Kit (Ventana) were used according to the manifacturer's recommendations for the visualization of the primary anti PD-L1 antibody.

Stained sections were scanned using Ventana iScan HT and scored based on percentage of positive tumor cells, irrespective of staining intensities, using a four-tiered system: 0=0%, 1=1-4%, 2=5-9%, 3=10-49%, 4=≥50%.

We considered as adequate cores that showed a neoplastic component ≥ 30%; therefore cores with an inferior percentage of neoplastic cells have been excluded.

**Figure 3:**
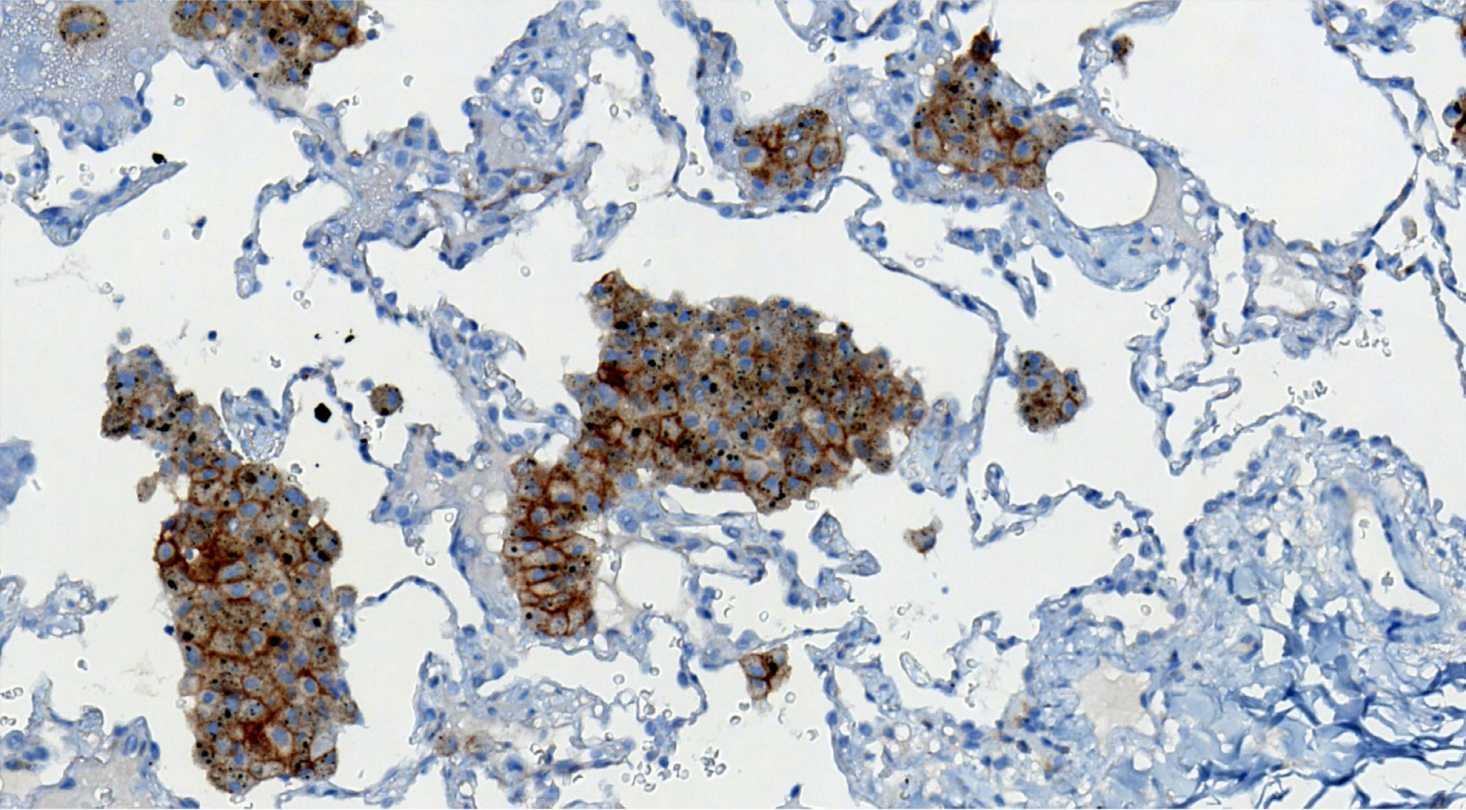
Macrophages were used as internal control

Macrophages were used as internal control in order to validate the adequacy of PD-L1 staining reaction (Figure 3).

### Statistical analysis

Statistical analysis was carried out using R version 3.2.3 [21] and R commander [22], including χ^2^, Cohen's κ coefficient of agreement and ROC analysis. *P* values < 0.05 were considered statistically significant.

## SUPPLEMENTARY MATERIALS TABLES


