# Correction: PD-L1 expression heterogeneity in non-small cell lung cancer: evaluation of small biopsies reliability

**DOI:** 10.18632/oncotarget.26475

**Published:** 2018-12-11

**Authors:** Enrico Munari, Giuseppe Zamboni, Marcella Marconi, Marco Sommaggio, Matteo Brunelli, Guido Martignoni, George J. Netto, Francesca Moretta, Maria Cristina Mingari, Matteo Salgarello, Alberto Terzi, Vincenzo Picece, Carlo Pomari, Gianluigi Lunardi, Alberto Cavazza, Giulio Rossi, Lorenzo Moretta, Giuseppe Bogina

**Affiliations:** ^1^ Department of Pathology, Sacro Cuore Don Calabria Hospital, Negrar, Italy; ^2^ Department of Pathology AOUI, University of Verona, Verona, Italy; ^3^ Department of Pathology, Pederzoli Hospital, Peschiera del Garda, Italy; ^4^ Department of Pathology, The University of Alabama at Birmingham, Birmingham, AL, USA; ^5^ Department of Laboratory Medicine, Sacro Cuore Don Calabria Hospital, Negrar, Italy; ^6^ Department of Experimental Medicine (DIMES), University of Genoa, Genoa, Italy; ^7^ Department of Nuclear Medicine, Sacro Cuore Don Calabria Hospital, Negrar, Italy; ^8^ Department of Thoracic Surgery, Sacro Cuore Don Calabria Hospital, Negrar, Italy; ^9^ Department of Oncology, Sacro Cuore Don Calabria Hospital, Negrar, Italy; ^10^ Department of Pulmonology, Sacro Cuore Don Calabria Hospital, Negrar, Italy; ^11^ Department of Pathology, Arcispedale S. Maria Nuova/IRCCS, Reggio Emilia, Italy; ^12^ Department of Pathology, Azienda USL Valle d'Aosta, Aosta, Italy; ^13^ Immunology Research Area, IRCCS Bambino Gesu Pediatric Hospital, Rome, Italy

**This article has been corrected:** In the Materials and Methods section, regarding the Tissue Microarray Construction, the diameter of the TMA core is incorrect. The proper value should be 1 mm, not 0.6 mm, as shown below:

**Tissue microarray construction**

For every case, all H&E stained slides were reviewed for diagnosis confirmation; one block was then selected for tissue microarray (TMA) construction. It has been demonstrated that TMAs containing at least 3 cores per case yield satisfactory agreement compared with whole section in lung cancer [14]. Therefore, for each block, 5 cores with a diameter of 1 mm were obtained randomly from the diverse areas of the tumor. Overall, 11 TMAs were built.

Original article: Oncotarget. 2017; 8:90123-90131. https://doi.org/10.18632/oncotarget.21485

