# Supplementary material for: PD-L1 expression heterogeneity in non-small cell lung cancer: evaluation of small biopsies reliability
Source: Oncotarget. 2017 Oct 4;8(52):90123–31. doi: 10.18632/oncotarget.21485 (PMC5685736; doi:10.18632/oncotarget.21485)
Supplement: Supplementary file 1 [file oncotarget-08-90123-s001.pdf]

PD-L1 expression heterogeneity in non-small cell lung cancer: evaluation of small biopsies reliability

SUPPLEMENTARY MATERIALS

Supplementary Table 1: PD-L1 expression within tissue cores of adenocarcinomas

A: PD-L1 cutoff ≥ 1%

		Tot	Neg			Positive		
**Number of evaluable cores**	**5**	128	82	11	6	8	2	19
	**4**	17	13	1	0	1	2	
	**3**	10	3	1	1	5		
	**2**	3	3	0	0			
	**1**	1	1	0				
		**159**	**0**	**1**	**2**	**3**	**4**	**5**
					**Number of positive cores**			

B: PD-L1 cutoff ≥ 50%

		Tot	Neg			Positive		
**Number of evaluable cores**	**5**	128	115	4	2	1	4	2
	**4**	17	16	0	0	0	1	
	**3**	10	6	0	0	4		
	**2**	3	3	0	0			
	**1**	1	1	0				
		**159**	**0**	**1**	**2**	**3**	**4**	**5**
					**Number of positive cores**			

Each cell shows number of adenocarcinoma cases (159) relative to the number of positive cores out of the number of available cores using ≥ 1% (A) and ≥ 50% (B) cutoffs.

Supplementary Table 2: PD-L1 expression within tissue cores of squamous cell carcinomas

A: PD-L1 cutoff ≥ 1%

		Tot	Neg	Positive				
Number of evaluable cores	**5**	50	27	3	3	5	2	10
	**4**	12	5	1	0	1	5	
	**3**	3	1	0	1	1		
	**2**	0	0	0	0			
	**1**	0	0	0				
		**65**	**0**	**1**	**2**	**3**	**4**	**5**
				Number of positive cores				

B: PD-L1 cutoff ≥ 50%

		Tot	Neg	Positive				
Number of evaluable cores	**5**	50	43	1	1	2	0	3
	**4**	12	10	1	0	1	0	
	**3**	3	3	0	0	0		
	**2**	0	0	0	0			
	**1**	0	0	0				
		**65**	**0**	**1**	**2**	**3**	**4**	**5**
				Number of positive cores				

Each cell shows number of squamous cell carcinomas (65) cases relative to the number of positive cores out of the number of available cores using ≥ 1% (A) and ≥ 50% (B) cutoffs.

Supplementary Table 3: ROC analysis for optimal number of cores

Statistics for 1% cut-off

# of cores	ROC area	Std. error	Chi2	df	Pr>chi2	Bonferroni Pr>chi2
**5 (standard)**	**1.0000**	**0.0000**				
1	0.8630	0.0263	27.1698	1	0.0000	0.0000
2	0.8973	0.0238	18.6207	1	0.0000	0.0000
3	0.9452	0.0184	8.8615	1	0.0029	0.0116
4	0.9863	0.0096	2.0282	1	0.1544	0.6176

Statistics for 50% cut-off

# of cores	ROC area	Std. error	Chi2	df	Pr>chi2	Bonferroi Pr>chi2
**5 (standard)**	**1.0000**	**0.0000**				
1	0.8571	0.0505	8.0000	1	0.0047	0.0187
2	0.8810	0.0476	6.2500	1	0.0124	0.0497
3	0.8810	0.0476	6.2500	1	0.0124	0.0497
4	0.9524	0.0328	2.1053	1	0.1468	0.5872

Overall, 191 cases had all 5 cores available and were used for the analysis.
We compared ROC curves obtained in this group of patients by one to four cores (cores were randomly ordered on the basis of their increasing number). Such analysis indicated a statistically significant differences between gold standard and number of cores < 4 for both 1% and 50% cutoff.
Four biopsies appear to be necessary to avoid misclassification.
